# Association of Bile Acid and Pepsin Micro-aspiration with Chronic Obstructive Pulmonary Disease Exacerbation

**Published:** 2019-01

**Authors:** Seyed-Mehdi Hashemi-Bajgani, Fatemeh Abbasi, Ahmad Shafahi, Rostam Yazdani, Mitra Samareh Fekri

**Affiliations:** 1 Department of Internal Medicine, School of Medicine, Afzalipour Hospital, Kerman University of Medical Sciences, Kerman, Iran; 2 Imam-Khomeini Hospital, Tehran University of Medical Sciences, Tehran, Iran; 3 Cardiovascular Research Center, Institute of Basic and Clinical Physiology Sciences, Kerman University of Medical Sciences, Kerman, Iran

**Keywords:** COPD, Aspiration, Bile acid, Pepsin

## Abstract

**Background:**

Chronic Obstructive Pulmonary Disease (COPD) is one of the most common chronic diseases all around the world. One of suggested risk factors for COPD is Gastroesophageal Reflux Disease (GERD). The aim of this study was investigation of the association between micro-aspiration of bile acid and pepsin with exacerbation attacks in COPD patients.

**Materials and Methods:**

The present study was a descriptive cross-sectional study. Fifty-two COPD patients were selected by simple sampling from patients referring to the Bessat Lung Clinic. Participants were divided into two groups of with and without COPD exacerbation history in the past year. The severity of the disease was determined based on the GOLD criteria (mild, moderate, severe and very severe). Then, all patients underwent bronchoscopy and the concentrations of bile acid and pepsin were compared in Broncho-Alveolar Lavage Fluid (BALF) of two groups.

**Results:**

The mean of bile acids in the group without COPD exacerbations was lower (27.38±3.26 μmol/Lit) than the group with COPD exacerbations (32.31±5.35 μmol/Lit) and this difference was not significant (P=0.436). The mean of pepsin in the first group was higher (118.46 ±15.44 ng/ml) than the second group (107.88±10.7 ng/ml) and this difference was also not significant (P=0.577).

**Conclusion:**

According to the results of this study, there is no association between disease severity and number of exacerbations with micro-aspiration of bile acid and pepsin in COPD patients.

## INTRODUCTION

World Health Organization (WHO) has defined Chronic Obstructive Pulmonary Disease (COPD) as a collection of pulmonary diseases that cause irreversible airflow limitation and chronic bronchitis, and emphysema and small airway disease are included in this group (1). In 2015, it was estimated that nearly three million deaths (5% of all deaths) in the world were attributed to COPD, and more than 90% of them occurred in low- and middle-income countries (2) and it was predicted that this disease will be the third leading cause of death in the world by 2020 (1).

Tobacco use is the primary risk factor for COPD and exposure to indoor and outdoor polluted air, as well as occupational dust, are other factors that cause this disease (2). The most important cause of death from COPD is acute exacerbations (3). Acute exacerbations occur in patients who have had COPD for long time and suddenly face an acute increase in one or more of the following symptoms: “cough frequency and severity; dyspnea; and change in the amount and/or character of sputum”. Patients with severe disease experience this condition two or three times within a year (4).

The mechanisms that affect acute exacerbations in COPD are not understood until now. According to the researches, more than 50% of the cases are due to viral and bacterial respiratory infections, 10% are due to environmental pollutions, and more than 30% are of unknown etiology (3,5).

Gastroesophageal Reflux Disease (GERD) is one of the risk factors that was proposed for COPD exacerbations and also among patients with GERD, upper respiratory symptoms are frequent (4,6). It has been demonstrated that Gastroesophageal Reflux (GER) plays an important role in causing extra-esophageal symptoms, including chronic bronchitis, bronchiectasis, diffuse panbronchiolitis, recurrent pneumonia, chronic cough, hoarseness, and asthma (7,8). Although available evidence suggests a relationship between COPD and GERD, but there are a few findings that show the impact of GER on the COPD and respiratory diseases (9,10). Micro-aspiration of gastric contents and bronchospasm due to vagal nerve stimulation following exposure of esophageal mucous membrane with gastric acid reflux are two major factors that explain the association between GER and the onset of pulmonary disease and its manifestations (11). A study showed a moderate relationship between sputum and exhaled breath condensate pepsin concentrations and it was suggested that exhaled breath condensate pepsin may be a useful noninvasive marker of pulmonary micro-aspiration, but it cannot be used for diagnosis of GERD (12).

Several studies have been done to investigate the effect of reflux on the increasing of COPD exacerbations (4,13–16) and a systematic review and meta-analysis showed GERD is a risk factor for COPD exacerbations (17). In a study in Canada, the results showed that in patients who developed obstructive bronchiolitis following lung transplantation, the prevalence of bile acid micro-aspiration is increased (15). Meanwhile, in another study the authors showed 37% of COPD patients had GER, and the number of COPD exacerbations in patients with GERD was reported two times more than GERD-free patients (2.3 compared to 1.6 in a year) (16).

Regarding the prevalence of GERD in patients with COPD, the aim of this study was to investigate the association of bile acid and pepsin micro-aspiration with COPD exacerbations in Kerman.

## MATERIALS AND METHODS

This was an analytical descriptive cross-sectional study. According to previous studies, the sample size was 52 patients. Participants were patients with COPD and their disease was confirmed by a pulmonary specialist; they were selected by simple sampling from patients referring to the Lung Clinic of Bessat (a clinic affiliated to Kerman University of Medical Sciences). Inclusion criteria was the COPD patients living in Kerman and exclusion criteria was the impossibility of carrying out bronchoscopy due to severe hypoxia, treatment-resistant cardiac arrhythmias, esophageal disease such as achalasia, esophageal cancer, stenosis, acute peptic ulcer and dissatisfaction for participating in the study. Participants were divided into two groups of 26 patients; one group had at least one COPD exacerbation (at least two weeks of the exacerbation must be passed for including the patient) including shortness of breath, cough, rate and change of sputum characteristics leading to emergency or hospital admission in the past year and the other group included patients without any COPD exacerbations. The first group included the C&D patients and the last one A&B patients based on GOLD criteria (ABCD assessment tool) (18). The clinical information on hoarseness, regurgitation, heartburn, dysphagia, and epigastric pain was collected at the time of clinical examination.

After giving the necessary explanations and obtaining consent, the samples were included and the severity of the disease in each patient was determined based on the GOLD criteria (mild, moderate, severe and very severe). According to the GOLD classification, all patients who had a forced expiratory volume in one second (FEV1) divided by forced vital capacity (FVC) ratio lower than 0.7 $\left(0.7>\frac{\text{FEV}1}{\text{FVC}}\right)$ after receiving two puff of a short-acting beta-agonist inhaled bronchodilators, is a COPD patient(18). When COPD was confirmed, the amount of FEV1 was used to determine the disease severity. FEV1 more than 80% is mild obstruction (stage I) and FEV1 between 50 to 80% is moderate obstruction (stage II), FEV1 between 30 and 50% is severe obstruction (stage III) and FEV1 less than 30% is very severe obstruction. Four factors were used to assess the severity of obstruction based on the BODE-index, including Body Mass Index (BMI), airway obstruction, FEV1, shortness of breath and exercise tolerance (6 minutes walking test) that provides more detailed information on the severity of obstruction and prognosis in comparison with FEV1 alone.

Then all patients underwent bronchoscopy and Bronchoalveolar Lavage Fluid (BALF) was obtained from the right middle lobe or right lower lobe, and about 60 ml of distilled water was introduced into the lung and 20 ml of the BALF sample was received. The BALF sample was immediately sent to the lab and stored at −70 to −80 centigrade. After sample collection, the amount of bile acids and pepsin of the BALF was measured by a biochemist, using the standard pepsin kit (ELISA Kit for pepsin) from the USCNK Company and the standard bile acid kit from the Company of Sigma. Independent sample t-test and Chi-square test were performed in SPSS 17.

## RESULTS

The mean age in groups of with and without COPD exacerbations history was 60.88±8.10 and 60.15±9.53 years, respectively. The large number (39 patients) (75%) of patients were male and without COPD exacerbation history (21 male patients in the past year). The BMI in groups of with and without COPD exacerbations history was 22.92±5.22 and 23.44±7.20, respectively and the number of participant who smoked was higher in the group that experienced at least one COPD exacerbation in the past year which was not significant either (P=0.50).

12 patients (46.2%) suffered from hoarseness in the first group (without COPD exacerbation) and 11 (42.3%) suffered from regurgitation in the second group (with COPD exacerbation) and these differences were not significant (P>0.05). The FEV1 based on the GOLD criteria showed that in both the first and the second groups with at least one COPD exacerbation during the past year, 13 of patients had moderate obstruction, and this was not statistically significant (P=0.964) (Table 1).

**Table 1.** Frequency of patient characteristics in the two groups of with and without COPD exacerbation

Variable		Without COPD exacerbations Frequency (%)	With COPD exacerbations Frequency (%)	Total Frequency (%)	P-value
**Mean age**		60.15±9.53	60.88±8.10	60.51	0.924
**BMI**		22.92±5.22	23.44±7.20	23.18	0.925
**Hoarseness**	Yes	12 (46.2)	7 (26.9)	19 (36.5)	0.125
	No	14 (53.8)	19 (73.1)	33 (63.5)	
**Regurgitation**	Yes	8 (30.8)	11 (42.3)	19 (36.5)	0.283
	No	18 (69.2)	15 (57.7)	33 (65.5)	
**Heartburn**	Yes	9 (34.6)	11 (42.3)	20 (38.5)	0.388
	No	17 (65.4)	15 (57.7)	32 (61.5)	
**Dysphagia**	Yes	7 (26.9)	4 (15.4)	13 (25)	0.100
	No	19 (73.1)	22 (84.6)	39 (75)	
**Epigastric pain**	Yes	7 (26.9)	8 (30.8)	15 (28.8)	0.500
	No	19 (73.1)	18 (69.2)	37 (71.2)	
**FEV1**	Mild	2 (7.7)	3 (11.5)	5 (9.6)	0.964
	Moderate	13 (50)	13 (50)	26 (50)	
	Severe	7 (26.9)	6 (23.1)	8 (15.4)	
	Very severe	4 (15.4)	4 (15.4)	8 (15.4)	

Although the mean of bile acids in the group without COPD exacerbation was lower (27.38±3.26 μmol/Lit) than the group with COPD exacerbation (32.31±5.35 μmol/Lit), this difference was not significant (P=0.436). The mean of pepsin in the first group was higher (118.46±15.44 ng/ml) than the second group (107.88±10.7 ng/ml) and this difference was also not significant (P=0.577). Also, the average number of cigarette packs per year in the first group was 360 packs and in the second group was 511 packs and this difference was not statistically significant (P=0.660) (Table 2).

**Table 2.** Comparing the mean of cigarette packs, bile acid and pepsin in the two groups of with and without COPD exacerbation

**Variable**	Without COPD exacerbations Frequency (%)	With COPD exacerbations Frequency (%)	P-value
**Cigarette packs (pack per year)**	36.0±4.5	51.41±6.26	0.066
**Bile acid (μmol/Lit)**	27.38±3.26	32.30±5.35	0.436
**Pepsin (ng/ml)**	118.46±15.44	107.88±10.77	0.577

There was no correlation between micro-aspiration markers with clinical symptoms, FEV1 and BODE index (Table 3).

**Table 3.** Correlation between micro-aspiration markers with clinical symptoms, FEV1 and BODE index

**Variable**	**Bile acid**		**Pepsin**	

	r	P-value	r	P-value
**FEV1**	−1.550	0.287	−0.30	0.834
**Hoarseness**	1.023	0.174	1.006	0.237
**Heartburn**	0.992	0.546	1.005	0.246
**Regurgitation**	0.992	0.527	1.003	0.546
**Dysphagia**	0.997	0.805	1.003	0.524
**Epigastric pain**	0.993	0.586	1.006	0.211

## DISCUSSION

It has been shown that GERD and micro-aspiration are common in many chronic respiratory diseases including COPD (19). In all studies, micro-aspiration of bile acid and pepsin was seen (the mean was 29.84±4.30 μmol/Lit) but we did not find any association between amount of micro-aspiration and number of COPD exacerbations.

Rogha et al.’s study showed that in COPD patients with GERD symptoms, exacerbations were more severe and hospitalization was increased rather than the patients without GERD symptoms (4). It seems that respiratory symptoms in COPD patients who suffered from GERD is aggregated because of direct stimulation of air ways and indirect stimulation of vagal nerve ending in esophagus by aspirated materials (20).

In normal conditions, finding bile acid and pepsin in lung is unexpected, and therefore no normal levels have been determined though Blondeau et al. in Belgium by investigating lung transplantation patients showed bile acid in BALF was found to be positive for about 50% of patients and it was not found in BALF of even one of the participant in the control group (21). D’Ovidio et al. in Canada showed that in lung transplant patients, a higher bile acid level in BALF was associated with increased early prevalence of bronchiolitis after transplantation. They also showed that increased concentration of bile acid in the BALF of these patients is associated with increased level of inflammatory factors in the alveoli, including neutrophils and IL-8 and diminished surfactant phospholipids. The patients also had more positive BAL cultures in terms of fungus and bacteria. They suggested that gastro-duodenal aspiration, by increasing inflammation and decreasing the intrinsic immunity, can cause pulmonary damage and the development of bronchiolitis after transplantation. In their study there was not any relationship between the serum level of bile acid and its level in BALF, and micro-aspiration may determine the bile acid levels in BALF (15). In the present study, we found bile acid and pepsin in BALF of all patients and with the mean of 29.84±4.30 μmol/Lit and 113.17±13.10 ng/ml, respectively.

The reason of increase in reflux and micro-aspiration in many chronic lung diseases is not well defined. In obstructive bronchiolitis following lung transplantation, causes include damage to the vagus nerve during transplantation, use of immunosuppressive drugs such as calcineurin inhibitors (cyclosporine and tacrolimus) and delay in gastric emptying (22–24).

In the present study, although the mean of bile acid and pepsin in the BALF of group without COPD exacerbation was lower than the group with COPD exacerbation, these differences were not significant and so, there was no significant relationship between GERD symptoms (hoarseness, heartburn, dysphagia, regurgitation and epigastric pain) and COPD exacerbations. In addition, there was no correlation between micro-aspiration markers with clinical symptoms, FEV1 and BODE index. These findings were different from other studies that have reported a direct relationship between GERD and COPD exacerbations. Khattab et al.’s study in Egypt showed that the number of COPD exacerbations in patients with GERD symptoms was significantly higher than patients without GERD symptoms (25). Mokhlesi et al. in the USA showed that the prevalence of GERD in patients with severe COPD was higher than controls (26). Likewise, the results of a study by Rascon-Aguilar et al. in USA showed that the rate of exacerbations of COPD was twice as high in patients with GERD symptoms compared to those without GERD symptoms (16).

The limitations of this study included loss of control group (because bronchoscopy is an invasive procedure), low sample size and limiting of GERD diagnosis to some questions.

## CONCLUSION

According to the results of this study, there was not any association between micro-aspiration of bile acid and pepsin with COPD exacerbations, severity of COPD, BODE-index and GERD symptoms.
